# Gesundheitskompetenz messen: Methoden und Instrumente zur Erfassung der allgemeinen Gesundheitskompetenz bei Erwachsenen

**DOI:** 10.1007/s00103-025-04010-y

**Published:** 2025-02-13

**Authors:** Robert Griebler, Thomas Link, Denise Schütze, Christa Straßmayr

**Affiliations:** 1 Kompetenzzentrum Gesundheitsförderung und Gesundheitssystem, Gesundheit Österreich GmbH, Stubenring 6, 1010 Wien, Österreich; 2 Abteilung Qualitätsmessung und Patientenbefragung, Gesundheit Österreich GmbH, Stubenring 6, 1010 Wien, Österreich

**Keywords:** Gesundheitskompetenz, Messung, Health Literacy Questionnaire (HLQ), HLS_19_-Q12, M‑POHL, Health literacy, Measurement, Health Literacy Questionnaire (HLQ), HLS_19_-Q12, M‑POHL

## Abstract

Die Messung von Gesundheitskompetenz (GK) begann in den 1990er-Jahren mit Instrumenten, die sich auf ein funktionales Verständnis von GK konzentrierten. Seither hat sich das Verständnis von GK und damit auch die Messung von GK weiterentwickelt. Für die Messung einer umfassenden allgemeinen GK werden in diesem Beitrag 2 besonders gut validierte Instrumente vorgestellt, der Health Literacy Questionnaire (HLQ) und der HLS_19_-Q12-Fragebogen.

Der HLQ umfasst 9 Skalen mit insgesamt 44 Items, die verschiedene GK-Aspekte in der Krankheitsbewältigung abdecken. Der HLQ wurde in zahlreichen Studien validiert und bisher in 47 Sprachen übersetzt. Er weist eine hohe Inhalts- und Kriteriumsvalidität auf und wird zum Beispiel im Rahmen des WHO European Action Network on Health Literacy for Prevention and Control of Noncommunicable Diseases und in der European Joint Action on Cardiovascular Diseases and Diabetes (JACARDI) eingesetzt. Auf Basis der 9 Skalen können GK-Profile zu Stärken und Herausforderungen erstellt werden.

Der HLS_19_-Q12 ist ein Kurzfragebogen zur Messung der allgemeinen GK und basiert auf dem HLS_19_-Q47. Er besteht aus 12 Items, wurde in mehr als 20 Ländern validiert und ist in mehr als 30 Sprachen übersetzt. Der HLS_19_-Q12 weist ebenfalls eine hohe Inhalts- und Kriteriumsvalidität auf und wird in den Health Literacy Surveys des WHO Action Network on Measuring Population and Organizational Health Literacy (M-POHL) und der European Joint Action Prevent Non-Communicable Diseases eingesetzt. Aus den 12 Items wird ein Gesamtwert berechnet, der in 4 GK-Stufen kategorisiert werden kann.

Um langfristig eine vergleichbare Datenbasis zu schaffen, wird empfohlen, diese beiden Instrumente in Studien, Evaluationen und für das Monitoring von GK zu verwenden.

## Abstract (en)

The measurement of health literacy (HL) began in the 1990s with instruments that focused on a functional understanding of HL. Since then, the understanding of HL and the measurement of HL have evolved. This article reviews two particularly well-validated instruments for measuring comprehensive general health literacy: The Health Literacy Questionnaire (HLQ) and the HLS_19_-Q12 questionnaire.

The HLQ consists of nine scales with a total of 44 items covering different HL aspects of coping with illness. The HLQ has been validated in numerous studies and translated into 47 languages. It has high content and criterion validity and is used, for example, in the WHO European Action Network on Health Literacy for Prevention and Control of Noncommunicable Diseases and in the European Joint Action on Cardiovascular Diseases and Diabetes (JACARDI). The nine scales can be used to create HL profiles of strengths and challenges.

The HLS_19_-Q12 is a short questionnaire to measure general HL and is based on the HLS_19_-Q47. It consists of 12 items, has been validated in more than 20 countries, and has been translated into more than 30 languages. The HLS_19_-Q12 also has high content and criterion validity and is used in the Health Literacy Surveys of the WHO Action Network on Measuring Population and Organizational Health Literacy (M-POHL) and the European Joint Action Prevent Non-Communicable Diseases. Based on the 12 items, a total score is calculated that can be categorized into four levels of HL.

In order to create a comparable database in the long run, it is recommended that these two instruments be used in studies, evaluations, and monitoring of HL.

## Gesundheitskompetenz messen – ein kurzer Abriss

Erste Instrumente zur Messung der Gesundheitskompetenz (GK) wurden in den 1990er-Jahren veröffentlicht, wie zum Beispiel der Test of Functional Health Literacy in Adults (TOFHLA) oder das Rapid Estimate of Adult Literacy in Medicine (REALM; [1–3]). Hierbei handelt es sich um Instrumente, die vor allem in den USA im Versorgungskontext eingesetzt wurden. Sie erfassen die GK von Patientinnen und Patienten durch verschiedene objektive Tests, die auf einem funktionalen Verständnis von GK beruhen und sich insbesondere auf das Verstehen schriftlicher medizinischer oder gesundheitsbezogener Informationen beziehen [4]. Später wurden diese Tests vereinzelt auch in allgemeinen Bevölkerungsstudien eingesetzt.

Seit diesen Anfängen hat sich jedoch das Verständnis von GK und damit auch die Messung von GK weiterentwickelt. Grob lassen sich 4 Entwicklungsrichtungen feststellen [5]:

- von der Fokussierung auf Krankheit und Krankheitsbewältigung hin zu Prävention, Gesundheitsförderung und einem umfassenden Gesundheitsverständnis [6, 7],
- von einem rein funktionalen Verständnis von GK (Lesen, Schreiben, Rechnen) hin zu interaktiven und kritischen Kompetenzen [8] bzw. Kompetenzen, die das Finden, Verstehen, Bewerten und Anwenden von Gesundheitsinformationen betreffen [7],
- von einem individualistischen hin zu einem relationalen Verständnis von GK, das GK nicht als rein individuelle Kompetenz, sondern als Interaktion zwischen individuellen Kompetenzen und den Anforderungen der Informations- und Angebotsumwelt begreift [9, 10], und
- von einem allgemeinen Verständnis von GK hin zu spezifischen Aspekten von GK (z. B. psychische GK, digitale GK, Impfkompetenz).

Ein erstes umfassendes Instrument zur Messung von GK, die Health Activities Literacy Scale (HALS), wurde in den USA entwickelt [6] und in den USA, Kanada und einigen europäischen Ländern (2003) eingesetzt [11–14], danach aber nicht mehr verwendet. Wie TOFHLA und REALM ist es ein leistungsorientiertes Instrument (Test) mit über 190 gesundheitsbezogenen Items.

In Europa begann die Messung der GK in der Bevölkerung in der Schweiz mit dem Swiss Health Literacy Survey (HLS-CH) im Jahr 2006 [15]. Der HLS-CH verwendete ein neues, hauptsächlich auf Selbsteinschätzung basierendes Befragungsinstrument, das, wie der HALS, verschiedene Dimensionen der GK berücksichtigte. Die in der Schweiz gemachten Erfahrungen und die durch die HLS-CH-Studie ausgelöste gesundheitspolitische Debatte haben dazu geführt, dass auch die Mitgliedsstaaten der Europäischen Union (EU) Daten zur GK ihrer Bevölkerung haben wollten. Infolge dessen wurde die erste europäische Gesundheitskompetenzstudie – European Health Literacy Survey (HLS-EU) – initiiert und in 8 Ländern durchgeführt [16]. Im Rahmen dieser Studie wurden ein umfassendes Modell allgemeiner GK [7] und ein neues Messinstrument, der European Health Literacy Questionnaire (HLS-EU‑Q; [17]), entwickelt. Die Ergebnisse der HLS-EU-Studie verdeutlichten, dass ein erheblicher Teil der Bevölkerung Schwierigkeiten hatte, gesundheitsrelevante Informationen zu nutzen. Darüber hinaus wurden ein sozialer Gradient in der GK und Zusammenhänge mit dem Gesundheitsverhalten, dem Gesundheitszustand und der Inanspruchnahme von Gesundheitsleistungen festgestellt [16, 18].

Das internationale Benchmarking der 8 HLS-EU-Länder erregte große Aufmerksamkeit und zeigte, dass GK als wichtiges gesundheitspolitisches Thema wahrgenommen wird, sobald Daten vorliegen. Die Ergebnisse der HLS-EU-Studie führten zu spezifischen gesundheitspolitischen Maßnahmen zur Verbesserung der GK, insbesondere in Österreich und Deutschland [19]. Die HLS-EU Ergebnisse wurden zudem in der WHO-Publikation „Health literacy: the solid facts“ berücksichtigt, in der auch die regelmäßige Durchführung von GK-Messungen empfohlen wird [20].

Diese Empfehlung führte 2018 zur Gründung des WHO Action Network on Measuring Population and Organizational Health Literacy (M-POHL^1^; [21]). Mit der zweiten europäischen Gesundheitskompetenzstudie – Health Literacy Survey 2019–2021 (HLS_19_) – hat M‑POHL dazu beigetragen, dass zur GK der europäischen Bevölkerung mittlerweile Daten aus 17 Ländern der WHO-Europa-Region vorliegen [22]. Im Rahmen der HLS_19_-Studie wurde das HLS-EU-Instrument zur Messung der allgemeinen GK angepasst (HLS_19_-Q47) und eine Kurzform mit 12 Items entwickelt (HLS_19_-Q12; [23]). Darüber hinaus wurden, dem internationalen Trend folgend, auch spezifische GK abgefragt (siehe Abschnitt „HLS_19_-Q12“; [22]).

Inzwischen gibt es eine Vielzahl von GK-Instrumenten, wobei der Trend zu einer immer differenzierteren Betrachtung für unterschiedliche Aspekte der GK, aber auch für unterschiedliche Bevölkerungsgruppen (z. B. Kinder, Jugendliche, ältere Menschen) ungebrochen scheint. Auch neue Themen, wie zum Beispiel die professionelle GK, werden aufgegriffen [24].

Angesichts dieser Vielschichtigkeit konzentriert sich der vorliegende Beitrag auf die Messung der allgemeinen GK bei Erwachsenen. Zunächst wird ein Überblick über die am häufigsten verwendeten Instrumente gegeben, ergänzt um Hinweise zur Messung spezifischer GK für darüber hinaus interessierte Leserinnen und Leser. In den nachfolgenden Abschnitten werden die derzeit am besten validierten Instrumente zur Messung einer umfassenden allgemeinen GK beschrieben: der Health Literacy Questionnaire (HLQ) und der HLS_19_-Q12-Fragebogen. Zum Schluss wird ein kurzes Fazit gezogen.

Ziel dieses Beitrags ist es, einen kompakten Überblick über die Methoden zur Messung der allgemeinen Gesundheitskompetenz bei Erwachsenen zu geben und 2 häufig verwendete und gut validierte Instrumente vorzustellen.

## Instrumente zur Messung der allgemeinen Gesundheitskompetenz

So unterschiedlich wie das Verständnis von GK sind auch die Messinstrumente, die für Forschung, Evaluation oder Monitoring zur Verfügung stehen. Seit der ersten Veröffentlichung eines Instruments zur Messung der funktionalen GK, dem REALM [1, 2], hat sich die Zahl der GK-Instrumente exponentiell vervielfacht. Der Health Literacy Tool Shed^2^, eine Online-Sammlung von GK-Instrumenten, bietet einen ersten Überblick. Gegenwärtig sind darin mehr als 200 Instrumente erfasst, wobei auch Übersetzungen und adaptierte Versionen enthalten sind. Nur ein Teil davon eignet sich zur Messung der allgemeinen GK.

Darüber hinaus haben zahlreiche systematische Übersichtsarbeiten die wachsende Zahl an GK-Instrumenten zusammengefasst, verglichen und bewertet. Allein zur Messung der allgemeinen GK gibt es mindestens 12 Übersichtsarbeiten [25–36]. Ohne eine genaue Zahl nennen zu können, stehen derzeit geschätzt mehr als 50 Instrumente zur Messung der allgemeinen GK zur Verfügung. Sie lassen sich in Instrumente unterteilen, die entweder auf einem funktionalen oder einem umfassenden Verständnis von GK beruhen, und in Instrumente, die performancebasiert (d. h. in Form von Leistungstests) oder erfahrungsbasiert (in Form von Selbstberichten) messen. Häufig findet sich eine Kombination aus „funktionalem Verständnis und Leistungsmessung“ und „umfassendem Verständnis und Selbstbericht“. Zu den am häufigsten genutzten Instrumenten in der Kategorie „funktionales Verständnis und performancebasiert“ gehören der TOFHLA, REALM und NVS (Newest Vital Sign; [33]), während in der Kategorie „umfassendes Verständnis und erfahrungsbasiert“ der HLQ [37] und der HLS-EU-Q47 [17], seine Kurzformen (HLS-EU-Q16 und HLS-EU-Q6; [38, 39]) und die adaptierte Kurzform HLS_19_-Q12 [23] zu nennen sind. Die beiden letztgenannten Instrumente (HLQ und HLS-EU-Q) sind laut einer aktuellen Übersichtsarbeit [35] auch die derzeit am besten validierten Instrumente zur Messung einer umfassenden allgemeinen GK.

## Exkurs: Instrumente zur Messung spezifischer Gesundheitskompetenzen

Neben den Instrumenten zur Messung der allgemeine GK gibt es mittlerweile auch zahlreiche Instrumente, die sich auf spezifische Gesundheitskompetenzen bzw. spezifische Aspekte der GK beziehen. Es handelt sich hier um:

- GK-Instrumente, die sich auf Patientinnen und Patienten mit bestimmten Erkrankungen beziehen (z. B. Atemwegserkrankungen, Herz-Kreislauf-Erkrankungen, Diabetes, psychische Erkrankungen; [40–49]) oder auf die Prävention übertragbarer Erkrankungen (Impfkompetenz; [50]),
- lebensstilbezogene Instrumente (z. B. zur Ernährungskompetenz oder zur bewegungsbezogenen GK; [51–53]),
- Instrumente zur digitalen GK/eHealth Literacy [54–58] oder
- Instrumente für bestimmte Altersgruppen (Kinder, Jugendliche und Ältere; [59–64]).

Entsprechende Übersichtsarbeiten werden bei den einzelnen Themenclustern referenziert. Darüber hinaus gibt es noch Übersichtsarbeiten, die sich mit spezifischen Formen der Datenerhebung [54, 55, 65] oder unterschiedlichen methodischen Zugängen in der GK-Messung befassen [66].

## Health Literacy Questionnaire (HLQ)

Der HLQ ist eines der beiden am besten validierten Instrumente zur Messung einer umfassenden allgemeinen GK und basiert auf der GK-Definition der WHO, wie sie im Health Promotion Glossary 1998 veröffentlicht wurde [67, 68]. Sie verweist auf die kognitiven und sozialen Fähigkeiten, die die Motivation und die Möglichkeiten des Einzelnen bestimmen, auf Informationen zuzugreifen, sie zu verstehen und zu nutzen, um die eigene Gesundheit zu fördern und zu erhalten. Basierend auf einem induktiven Ansatz zur Instrumentenentwicklung [69] wurde von einem Team um Richard Osborne (heute Swinburne University of Technology, Australien) auf der Grundlage einer in Workshops erarbeiteten Sammlung relevanter Aspekte von GK ein multifaktorielles Messinstrument entwickelt, das aus 9 Faktoren („domains“) mit jeweils 4 bis 5 Items (insgesamt 44) besteht. Mit seinen 9 Skalen hat das Instrument vornehmlich den Bereich der Krankheitsbewältigung im Fokus [70]:

1. sich von Gesundheitsdienstleistern verstanden und unterstützt fühlen („feeling understood and supported by healthcare providers“),
2. ausreichende Informationen haben, um meine Gesundheit zu managen („having sufficient information to manage my health“),
3. aktiv meine Gesundheit managen („actively managing my health“),
4. soziale Unterstützung für Gesundheit („social support for health“),
5. Gesundheitsinformationen bewerten („appraisal of health information“),
6. Fähigkeit, sich aktiv mit Leistungserbringern auseinanderzusetzen („ability to actively engage with healthcare providers“),
7. sich im Gesundheitssystem zurechtfinden („navigating the health system“),
8. Fähigkeit, gute Gesundheitsinformationen zu finden („ability to find good quality health information“),
9. Gesundheitsinformationen ausreichend gut verstehen, um zu wissen, was zu tun ist („understanding health information well enough to know what to do“).

Die Items der Faktoren 1 bis 5 sind auf einer 4‑stufigen Ratingskala („strongly disagree“ bis „strongly agree“) und die Items der Faktoren 6 bis 9 auf einer 5‑stufigen Ratingskala („cannot do“ bis „very easy“) zu beantworten [71]. Aufgrund der Länge des Instruments wird in einigen Studien nur eine Auswahl der 9 Skalen verwendet (z. B. Bo et al. [72]; Simpson et al. [73]).

Für jeden Faktor wird ein Score errechnet. Die berechneten Scores sind Summenscores mit unterschiedlichen Wertebereichen je nach Anzahl der Items und Ratingskala. Von der Berechnung eines Gesamtscores wird mit Hinweis auf die Mehrdimensionalität des zugrunde liegenden Konzepts bzw. des Instruments abgesehen. Es werden zudem keine GK-Niveaus („levels“) berechnet. Die Idee einer Einteilung der Bevölkerung in Personen mit guter oder schlechter GK wird grundsätzlich zurückgewiesen. Stattdessen werden auf Basis der 9 Scores GK-Profile erstellt. Der Fokus liegt dabei auf der Kombination von Stärken und Herausforderungen, um darauf aufbauend gezielte Interventionen zu entwickeln, z. B. im Rahmen des Ophelia-Prozesses (Optimising Health Literacy and Access; [74]).

Der HLQ wurde in zahlreichen Studien in verschiedenen Sprachen und Kontexten eingesetzt^3^ bzw. validiert [35]. Derzeit liegt das Instrument in 47 Sprachen übersetzt bzw. kulturell adaptiert vor (auch in deutscher Sprache [70]). 4 weitere Übersetzungen sind in Arbeit. Das Instrument weist eine gute Inhalts- und Kriteriumsvalidität auf, wobei letztere für gesundheitsrelevante Verhaltensweisen, Gesundheitsindikatoren und die Inanspruchnahme professioneller Gesundheitsdienste nachgewiesen wurde [69]. Seine faktorielle Validität wurde in konfirmatorischen Faktorenanalysen (CFA) und vereinzelt auch in Rasch-Analysen bestätigt. Osborne et al. [69] validierten die angenommene Faktorenstruktur mit einem 9‑Faktoren-CFA-Modell und stellten eine gute Modellanpassung fest (CFI = 0,936, TLI = 0,930 und RMSEA = 0,076)^4^. Nolte et al. [70] berichten für die deutsche Übersetzung vergleichbare Werte (CFI = 0,990, RMSEA = 0,048). Mit Cronbachs-Alpha-Koeffizienten um oder über 0,8 weisen alle 9 Skalen zudem eine gute interne Konsistenz auf [69, 70]. In einer weiterführenden Studie [75] wurde außerdem die Eindimensionalität der 9 Skalen mittels Rasch-Analysen bestätigt. Allerdings stellten die Autorinnen und Autoren auch inhaltliche Überschneidungen fest, die sie jedoch als unkritisch beurteilten. Hinweise auf eine zumindest teilweise Überlappung einzelner Faktoren finden sich auch in Osborne et al. [37] sowie Nolte et al. [76], sodass unter Umständen ein zugrunde liegender, übergeordneter Faktor angenommen werden kann. Dies muss allerdings in weiterführenden Analysen erst final geklärt werden [77]. Die Konvergenzvalidität des HLQ wurde mit Instrumenten zur Messung der funktionalen GK (TOFHLA, NVS) untersucht [78], die allenfalls schwach mit den HLQ-Skalen korrelieren. Lediglich Faktor 5 (Gesundheitsinformationen bewerten) korreliert mit ρ = −0,28 etwas stärker mit dem NVS, ebenso Faktor 8 (Gesundheitsinformationen finden) und Faktor 9 (Gesundheitsinformationen verstehen) mit ρ = 0,23 bzw. ρ = 0,32 (Spearman-Korrelation) mit dem TOFHLA Reading. In einer rezenten Studie [79] wurden zudem Zusammenhänge mit dem HLS_19_-Q12 (siehe nächster Abschnitt) ermittelt. Die 9 HLQ-Skalen korrelieren positiv im moderaten Bereich mit dem HLS_19_-Q12-Score (zwischen r = 0,24 und r = 0,42; Pearson-Korrelation). Hierbei gilt es jedoch zu beachten, dass sich der HLQ und der HLS_19_-Q12 inhaltlich nur in Teilen überschneiden.

Neuübersetzungen des Instruments müssen gemäß der Translation Integrity Procedure [80] validiert werden, die sowohl eine qualitative Validierung mittels kognitiver Interviews als auch eine statistische Validierung empfiehlt. Das Instrument darf außerdem nur mit einer Genehmigung der Swinburne University of Technology verwendet werden. Die Nutzung ist zwar für nicht geförderte wissenschaftliche Forschung sowie für gemeinnützige und nichtkommerzielle Projekte und Organisationen kostenlos, jedoch an Bedingungen geknüpft, wie z. B. das Verbot der Veröffentlichung des verwendeten Fragebogens, was für einige Anwendungsbereiche ein K.-o.-Kriterium darstellen könnte. Ansonsten ist die Nutzung des Instruments mit Kosten verbunden. Der HLQ ist für verschiedene Erhebungsmethoden geeignet (persönliche Interviews, Telefoninterviews, Onlinebefragungen, Paper-Pencil-Befragungen) und einfach in der Handhabung. Die durchschnittliche Bearbeitungszeit beträgt 7–8 min. Der HLQ wird verstärkt in National Health Literacy Demonstration Projects on Non-communicable Diseases (NCDs) im Rahmen des WHO European Action Network on Health Literacy for Prevention and Control of NCDs [81] und in der European Joint Action on Cardiovascular Diseases and Diabetes^5^ (JACARDI) eingesetzt.

## HLS_19_-Q12

Der HLS_19_-Q12, ein Kurzfragebogen zur Messung der allgemeinen GK [23], stellt das Herzstück der eingangs erwähnten M‑POHL-HLS_19_-Studie und auch der Folgestudie HLS_24_ (Health Literacy Survey 2024–2026) dar. Er wurde auf der Basis des HLS_19_-Q47 [22] entwickelt, einer adaptierten Version des HLS-EU-Q47 [17], der zusammen mit seinen Kurzformen (HLS-EU-Q16 und HLS-EU-Q6; [19]) zu den am besten validierten Instrumenten zur Messung einer umfassenden allgemeinen GK gehört [35].

Dem HLS_19_-Q12/-Q47 und seinen Vorgängerversionen liegt ein umfassendes GK-Verständnis zugrunde [7]. Es umfasst das Wissen, die Motivation und die Fähigkeiten von Menschen, relevante Gesundheitsinformationen zu finden, zu verstehen, zu bewerten und anzuwenden, um im Alltag Urteile und Entscheidungen in den Bereichen Krankheitsbewältigung, Krankheitsprävention und Gesundheitsförderung zu treffen, die zu mehr Gesundheit und Lebensqualität beitragen [16]. In eine 3 × 4-Matrix übertragen ergeben sich daraus 12 Zellen, die relevante Subdimensionen einer allgemeinen GK definieren (Tab. 1).

**Tab. 1:** Subdimensionen von Gesundheitskompetenz

	Informationen finden	Informationen verstehen	Informationen beurteilen	Informationen anwenden
Krankheitsbewältigung	Informationen über Krankheitsbewältigung finden	Informationen über Krankheitsbewältigung verstehen	Informationen über Krankheitsbewältigung beurteilen	Informationen über Krankheitsbewältigung anwenden
Prävention	Informationen über Prävention finden	Informationen über Prävention verstehen	Informationen über Prävention beurteilen	Informationen über Prävention anwenden
Gesundheitsförderung	Informationen über Gesundheitsförderung finden	Informationen über Gesundheitsförderung verstehen	Informationen über Gesundheitsförderung beurteilen	Informationen über Gesundheitsförderung anwenden

Aus [7], Lizenz Creative Commons Attribution 2.0 Generic [93], die Tabelle wurde für die vorliegende Publikation durch die Autorinnen und Autoren übersetzt

In der Definition und in der Matrix nicht berücksichtigt, aber den HLS-EU- und HLS_19_-Instrumenten inhärent ist der relationale Charakter von GK [10]. Demnach entsteht GK aus dem Zusammenspiel individueller Kompetenzen mit den Anforderungen der Informations- und Angebotsumwelt und der daraus resultierenden Motivation [9]. Die HLS-EU- und HLS_19_-Instrumente fragen daher nach Schwierigkeiten bei der Ausführung von GK-Aufgaben [17, 23].

Der HLS_19_-Q12 ist eine Kurzform des HLS_19_-Q47. Er operationalisiert alle Zellen der 3 × 4-Matrix und umfasst 12 Items, die im Frageformat formuliert sind, um die Befragten direkt anzusprechen und das Verständnis zu erleichtern. Die unpersönliche Frageformulierung („Wie leicht oder schwer, würden Sie sagen, ist es …“) lädt zudem dazu ein, auch über erwartete, aber nicht erlebte Schwierigkeiten zu berichten. Die Antwortkategorien sind als eine voll verbalisierte 4‑stufige Ratingskala mit einer symmetrischen Anzahl von Antwortmöglichkeiten ausgeführt (von „sehr einfach“ bis „sehr schwierig“), um Tendenzen zur Mitte oder ausweichende Antworten („weiß nicht“) zu vermeiden. Dies ermöglicht auch eine einfache und interpretierbare Dichotomisierung der Antwortkategorien. Die einzelnen Items geben konkrete Hinweise auf bestehende Schwierigkeiten in der Bevölkerung. Gleichzeitig kann aus den einzelnen Fragen ein Score errechnet werden. Darüber hinaus wird eine Kategorisierung des Scores in 4 GK-Stufen vorgeschlagen, die den „match“ bzw. „mismatch“ zwischen individuellen Kompetenzen und situativen Anforderungen beschreiben und eine einfache proportionale Charakterisierung der GK in der Bevölkerung anhand der Kategorien inadäquat, problematisch, ausreichend und ausgezeichnet ermöglichen.

Der HLS_19_-Q12 wurde im Rahmen der M‑POHL-HLS_19_-Studie für 17 Länder validiert [23] und weist – ebenso wie der HLS-EU-Q47 und seine Kurzformen – eine gute Inhalts- und Kriteriumsvalidität auf, wobei letztere für gesundheitsrelevante Verhaltensweisen, Gesundheitsindikatoren und die Inanspruchnahme professioneller Gesundheitsdienste nachgewiesen wurde [22, 23]. Seine faktorielle Validität wurde durch konfirmatorische Faktorenanalysen (CFA) und vereinzelt auch durch Rasch-Analysen [23, 82] auch über HLS_19_ hinaus [79, 83, 84] bestätigt. Das länderweise berechnete einfaktorielle CFA-Modell zeigt in allen Ländern (darunter auch Deutschland und Österreich) eine gute Modellanpassung (CFI ≥ 0,97, TLI ≥ 0,96 und in 16 von 17 Ländern RMSEA-Werte ≤ 0,07). Mit Cronbachs-Alpha-Koeffizienten über 0,8 ist zudem eine gute interne Konsistenz gegeben [23]. Die Konvergenzvalidität des HLS_19_-Q12 wurde bisher nur in der Studie von Liu et al. [79] im Vergleich zum HLQ untersucht. Dabei korreliert der HLS_19_-Q12-Score positiv im moderaten Bereich (zwischen r = 0,24 und r = 0,42; Pearson-Korrelation) mit den 9 HLQ-Skalen. Allerdings ist zu beachten, dass sich der HLS_19_-Q12 und der HLQ inhaltlich nur teilweise überschneiden.

Die Kürze des Fragebogens ermöglicht einen flexiblen Einsatz sowohl in Studien als auch in Evaluationen und eignet sich hervorragend für Monitoringzwecke. Sie ermöglicht auch, neben der allgemeinen GK weitere Aspekte der GK zu berücksichtigen. Im Rahmen der HLS_19_-Studie wurden daher optionale Fragensets entwickelt und angeboten. Diese basieren auf den gleichen methodischen Prinzipien wie der HLS_19_-Q12 und können daher hinsichtlich ihrer Ergebnisse mit der allgemeinen GK verglichen werden. So wurden im HLS_19_ Daten zur digitalen GK [85], zur kommunikativen GK [86], zur navigationalen GK [87] und zur impfbezogenen GK [88] erhoben. In Kombination mit der allgemeinen GK ermöglichen diese spezifischen Instrumente eine umfassende Analyse der GK in der Bevölkerung [22]. Für den M‑POHL Health Literacy Survey 2024–2026 (HLS_24_) wurden die HLS_19_-Instrumente zur Messung spezifischer GK weiterentwickelt und um ein Instrument zur psychischen GK ergänzt.

Der HLS_19_-Q12 wurde bisher in mehr als 30 Sprachen übersetzt, wobei weitere Übersetzungen in Arbeit sind. Er ist für verschiedene Erhebungsmethoden geeignet (persönliche Interviews, Telefoninterviews, Onlinebefragungen, Paper-Pencil-Befragungen) und einfach in der Anwendung. Die durchschnittliche Bearbeitungszeit beträgt etwa 2–3 min. Der HLS_19_-Q12 steht für Forschung, Evaluationen und für die nichtkommerzielle Nutzung, z. B. durch Gesundheitsdienste, zur Verfügung und wird im Rahmen der M‑POHL Health Literacy Surveys und der European Joint Action Prevent Non-Communicable Diseases^6^ eingesetzt. Der HLS_19_-Q12 kann kostenlos über das M‑POHL International Coordination Center bezogen werden.^7^

## Schlussbetrachtung

GK ist eine zentrale Determinante von Gesundheit, ein wichtiger Hebel für mehr gesundheitliche Chancengerechtigkeit und eine Voraussetzung für selbstbestimmte Entscheidungen in Gesundheitsfragen [20, 89, 90]. Eine geringe GK geht mit einem ungünstigen Gesundheits‑, Risiko-, Präventions- und Krankheitsverhalten, einem schlechteren Gesundheitszustand und einer höheren Sterblichkeit sowie einer inadäquaten und erhöhten Inanspruchnahme des Gesundheitssystems und höheren Kosten in der Krankenbehandlung einher [20, 91]. Sie ist in der Bevölkerung ungleich verteilt [22] und wird infolge ökologischer und gesellschaftlicher Dynamiken (Klimawandel, Naturkatastrophen, Pandemien, alternde Gesellschaft, Digitalisierung und steigende Gesundheitskosten) immer wichtiger.

Im Gegensatz zu anderen sozialen Determinanten von Gesundheit ist die GK beeinflussbar, sei es durch Interventionen zur Erhöhung der individuellen Kompetenzen oder durch Maßnahmen, die darauf abzielen, die Anforderungen zur Nutzung gesundheitsrelevanter Informationen und Angebote zu reduzieren [92]. Daten zur GK tragen dazu bei, GK auf die (politische) Agenda zu setzen. In Österreich hat beispielsweise das schlechte Abschneiden in der EU-HLS-Studie dazu geführt, dass der GK ein eigenes Gesundheitsziel gewidmet (gesundheitsziele-oesterreich.at), die Österreichische Plattform GK gegründet (oepgk.at) und die GK nachhaltig in der Gesundheitsreform (Zielsteuerung-Gesundheit) verankert wurde. Die Messung von GK ermöglicht zudem, Herausforderungen und Zielgruppen zu identifizieren und Entwicklungen zu beobachten. Dies ermöglicht, die Planung und Umsetzung gezielter und zielgruppenspezifischer GK-Maßnahmen.

Die Auswahl eines geeigneten Messinstruments für Forschung, Evaluation oder Monitoring orientiert sich zunächst am Verständnis von GK. Hier zeigt sich, dass vor allem Instrumente, die ein breites Verständnis von GK operationalisieren, in den Fokus gerückt sind. Hierbei handelt es sich vor allem um Selbsteinschätzungsinstrumente. Im Gegensatz zu performanceorientierten Instrumenten, die auf die funktionale GK fokussieren, erfassen sie alle Aspekte einer umfassenden GK und berücksichtigen teilweise auch das relationale Verständnis von GK. Das oft kritisierte „Rauschen“ in Selbstberichtsdaten verweist in der Regel auf die Selbstwirksamkeit der Befragten und steht der Nützlichkeit der Daten und Ergebnisse nicht entgegen, da Selbsteinschätzungen das eigene Handeln bestimmen und damit alltagsrelevant werden. Bei der Interpretation der Ergebnisse gilt es aber, diese Selbsteinschätzungseffekte zu berücksichtigen.

Die Vielzahl der zur Verfügung stehenden Instrumente ermöglicht die gezielte Auswahl eines „passenden“ GK-Instruments, erschwert aber die Bereitstellung einer vergleichbaren Datenbasis zur Generierung von Evidenz. Es empfiehlt sich daher, in Studien, Evaluationen oder für Monitoringzwecke Instrumente zu verwenden, die bereits gut etabliert sind. In Bezug auf die allgemeine GK sind dies der HLS_19_-Q12 bzw. -Q47 und diesbezügliche Vorgängerinstrumente sowie der HLQ. Beide Instrumente sind gut validiert, weitverbreitet und liegen auch in deutscher Sprache vor. Während der HLS_19_-Q12 bzw. -Q47 eine starke Public-Health-Orientierung aufweist, fokussiert der HLQ stärker auf die Krankenbehandlung.

Die Tatsache, dass beide Instrumente bereits in zahlreichen Sprachen verfügbar sind und in vielen Ländern eingesetzt wurden, ermöglicht darüber hinaus ihren Einsatz in mehrsprachigen Studien und ggf. den Vergleich kleinerer regionaler Studien oder Evaluationsstudien mit repräsentativen nationalen Daten.

## Footnotes

1 https://m-pohl.net; zugegriffen am 26.11.2024.

2 https://www.tuftsmedicine.org/research-clinical-trials/research-institutes-research-department/center-health-literacy-research-and-practice/health-literacy-tool-shed; zugegriffen am 26.11.2024.

3 Für einen Überblick siehe: HLQ Licences by Country [Zugegriffen am 16.09.2024].

4 CFI: Comparative Fit Index, TLI: Tucker-Lewis Index, RMSEA: Root Mean Square Error of Approximation.

5 https://jacardi.eu; zugegriffen am 26.11.2024.

6 https://preventncd.eu; zugegriffen am 26.11.2024.

7 https://m-pohl.net/HLS19Instruments; zugegriffen am 26.11.2024.
